# Atypical response regulators expressed in the maize endosperm transfer cells link canonical two component systems and seed biology

**DOI:** 10.1186/1471-2229-10-84

**Published:** 2010-05-07

**Authors:** Luís M Muñiz, Joaquín Royo, Elisa Gómez, Gaelle Baudot, Wyatt Paul, Gregorio Hueros

**Affiliations:** 1 Departamento de Biología Celular y Genética, Universidad de Alcalá, Campus Universitario, Carretera de Madrid-Barcelona km 33.600, 28871 Alcalá de Henares (Madrid), Spain; 2 Biogemma SAS, 24 Avenue des Landais 63, 170 Aubière, France

## Abstract

**Background:**

Two component systems (TCS) are phosphotransfer-based signal transduction pathways first discovered in bacteria, where they perform most of the sensing tasks. They present a highly modular structure, comprising a receptor with histidine kinase activity and a response regulator which regulates gene expression or interacts with other cell components. A more complex framework is usually found in plants and fungi, in which a third component transfers the phosphate group from the receptor to the response regulator. They play a central role in cytokinin mediated functions in plants, affecting processes such as meristem growth, phyllotaxy, seed development, leaf senescence or tissue differentiation. We have previously reported the expression and cellular localization of a type A response regulator, *ZmTCRR-1*, in the transfer cells of the maize seed, a tissue critical for seed filling and development, and described its regulation by a tissue specific transcription factor. In this work we investigate the expression and localization of other components of the TCS signalling routes in the maize seed and initiate the characterization of their interactions.

**Results:**

The discovery of a new type A response regulator, *ZmTCRR-2*, specifically expressed in the transfer cells and controlled by a tissue specific transcription factor suggests a previously unknown role for TCS in the biology of transfer cells. We have characterized other canonical TCS molecules, including 6 histidine kinases and 3 phosphotransfer proteins, potentially involved in the atypical transduction pathway defined by *ZmTCRR-1 *and *2*. We have identified potential upstream interactors for both proteins and shown that they both move into the developing endosperm. Furthermore, *ZmTCRR-1 *expression in an heterologous system (*Arabidopsis thaliana*) is directed to xylem parenchyma cells, probably involved in transport processes, one of the major roles attributed to the transfer cell layer.

**Conclusions:**

Our data prove the expression of the effector elements of a TCS route operating in the transfer cells under developmental control. Its possible role in integrating external signals with seed developmental processes is discussed.

## Background

Two component signal transduction systems (TCS) in plants were first described in 1996 and rapidly ascribed to the signalling of a number of external outputs, including phosphate and nitrogen availability [1,2] and stress [3], usually through hormonal compounds [4-6]. More recently, related molecules have been linked to circadian regulation [7]. The full TCS structure in plants involves the perception of an external signal through membrane-bound histidine kinases (HK), dimerization and trans-phosphorylation of the receptor and subsequent transfer of a phosphate group among alternate histidine and aspartic residues in the kinase receptor, intermediate phosphotransfer proteins (HP) and response regulators (RR), respectively. The conformational change induced by phosphorylation modifies the response regulator activity, either in its capacity to interact with other downstream proteins (in the case of type-A RR) or by modification of its function as a transcriptional regulator (in the case of type-B RR) [8,9]. A wide array of physiological processes is regulated through phosphorelay routes, including senescence, meristem activity both in shoots and roots [10,11], circadian mechanisms [7] and tissue differentiation [12]. This multiplicity of tasks and the general ubiquity of most TCS molecules underscore the probable functional redundancy of elements within the system and the need for additional, external-to-the-route regulatory mechanisms.

Transfer cells (TC) occupy the base of the maize endosperm, directly in front of the maternal vascular terminals, and represent a modified surface, specialized in the uptake of nutrients from the apoplastic discontinuity between the mother plant and the seed [13,14]. This transport specialization, similarly to other transfer cells and transport-associated tissues elsewhere in the plant, involves the development of a highly invaginated basal surface, which presents flange wall ingrowths closely covered by cell membrane, abundant mitochondria and endoplasmic reticulum (ER) [15,16]. Additionally to nutrients, the basal endosperm transfer layer (BETL) cells receive environmental and developmental signals through the pedicel, which affect their own differentiation and function, and the proper growth and filling of the kernel. In particular, sugars and the hormones abscisic acid (ABA) and cytokinin have been shown to reach the mother-seed interface in high concentrations and affect the kernel development significantly [17-19], suggesting that transfer cells possibly play a role in the interpretation and further transmission of those signals. In recent years a number of transfer cell specific genes have been described [20-22] including a MYB-related transcription factor, *ZmMRP-1*, which controls the expression of several of these genes [23] through interaction with specific DNA motifs in their promoters [24]. In a previous paper we reported the isolation of *ZmTCRR-1*, a type-A response regulator exclusively expressed in the transfer cell layer of the maize caryopsis during early development, and transcriptionally controlled by *ZmMRP-1*. Remarkably, the ZmTCRR-1 protein is exported from the transfer cells into the endosperm, where it can be detected at later developmental stages [25]. In this work we describe a second transfer cell-specific response regulator, related in sequence to *ZmTCRR-1*, and their interaction with known TCS molecules in maize. Immunolocation and expression data within the seed for several proteins related to this transduction system support a role for the basal endosperm transfer cells in the perception of mother plant-borne signals and its transmission to the developing grain, with the hormone-insensitive, *ZmMRP-1*-controlled response regulators *ZmTCRR-1 *and *ZmTCRR-2 *conferring specificity to an otherwise ubiquitous two component signalling route. We also obtained circumstantial evidence indicating that similar pathways operate in *Arabidopsis *in connection with transport-related tissues.

## Results

### Cloning of *ZmTCRR-2*

**Figure 1:**
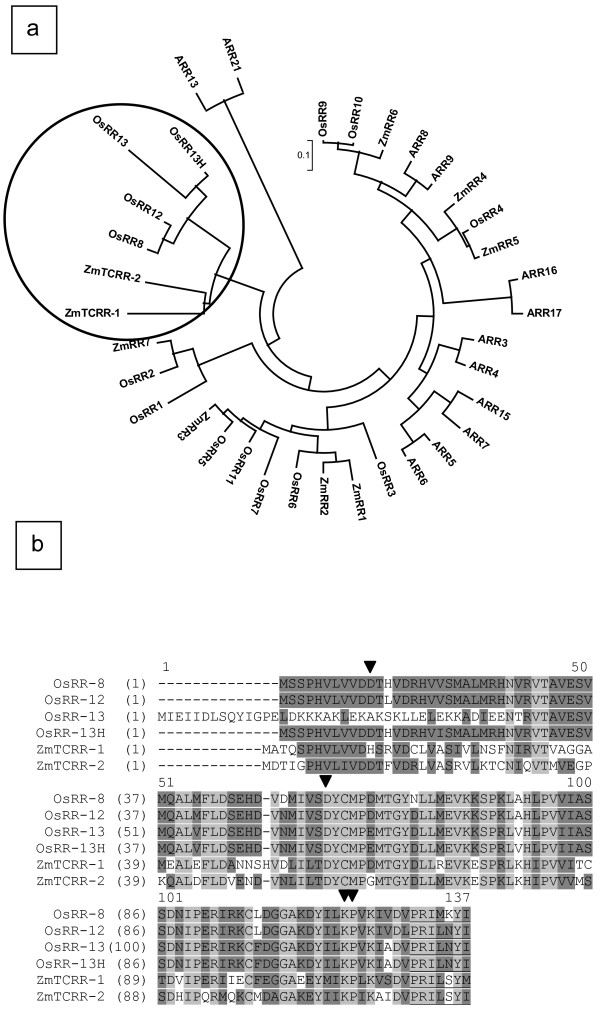
**Comparison of ZmTCRRs to plant type-A response regulators**. **a, **phylogenetic tree based on the protein sequences of type-A **r**esponse regulators from *Arabidopsis thaliana*, *Oryza sativa *and *Zea mays*. Note that OsRR13 and OsRR13H refer to different sequences named similarly in Ito et al. 2006 and Hirose et al. 2007, respectively. NCBI GI numbers for all sequences are supplied in Table 1. The alignment and distance calculations were performed using the MEGA 3.1 software [64] and the Neighbour-Joining algorithm with 1000 bootstrap iterations. The circle indicates the cluster where both ZmTCRRs are located together with four putative flower specific molecules from rice. **b, **alignment of ZmTCRRs and rice related proteins. The arrowheads indicate conserved residues forming the active site of response regulators, as indicated in the Conserved Domain Database (CDD) [65]. Conservative changes are shown on a dark background, identical residues on a light grey background. The common c-terminal motif specific for this cluster of proteins is underlined.

*ZmTCRR-2 *was identified in a BLAST search of a private cDNA collection using the coding sequence of *ZmTCRR-1*. BLAST searches in the maize genome sequence http://www.maizesequence.org revealed that *ZmTCRR-2 *is located on chromosome 1, and similarly to *ZmTCRR-1 *(located on chromosome 4), contains 5 exons with short untranslated extensions, and a coding region of 369 bp. The exon sizes and the splicing positions are similar in both genes, except for exon 3 which encodes an additional amino acid in *ZmTCRR-1*. A synteny analysis to rice and sorghum genomes using the tools in the maize database sequence http://www.maizesequence.org links both loci to a single chromosome of rice (chromosome 8) or sorghum (chromosome 7). The corresponding syntenic regions are, however, in different chromosome arms, suggesting that they may have originated in an intra-chromosomal duplication event predating the separation of these species around 50 million years ago [26]. This event would have been followed by genome reorganisation in *Zea mays *(additional file 1). A BLAST analysis of the CDS with the NCBI non redundant database http://www.ncbi.nlm.nih.gov shows homologies to known type-A response regulators from plants and bacteria. The alignment of the protein sequences with response regulators from rice, *Arabidopsis *and maize (NCBI GI numbers listed in Table 1) clusters ZmTCRR-1 and ZmTCRR-2 together with OsRR8, 12 and 13 [6] and OsRR13H [[27]; this locus is different to OsRR13 in [6], and the suffix "H" has been added here for clarification] (Figure 1a). These proteins (with a total sequence similarity of 87.5%) share a reduced size even for type-A standards (121-137 aa), and a common C-terminal motif (PRI [L,M] [S,N,K]Y [I,M]), which is in fact the longest similar stretch, not present in other RR sequences. Contrary to ZmTCRR-1 [25], ZmTCRR-2 possesses all the canonical amino acids described to be critical for response regulator activity [8] (Figure 1b).

**Table 1:** Type A response regulators identified in *Arabidopsis thaliana*, *Oryza sativa *and *Zea mays*.

PROTEIN	NCBI GI number
**ARR3**	15218957

**ARR4**	15218550

**ARR5**	15228338

**ARR6**	15242000

**ARR7**	15221927

**ARR8**	15227325

**ARR9**	15230202

**ARR13**	50400707

**ARR15**	15222035

**ARR16**	15226732

**ARR17**	18410485

**ARR21**	50400666

**OsRR1**	115458564

**OsRR2**	115446725

**OsRR3**	115450024

**OsRR4**	115442281

**OsRR5**	115459496

**OsRR6**	115461262

**OsRR7**	71273487

**OsRR8**	115476168

**OsRR9**	115484121

**OsRR10**	87116390

**OsRR11**	115447443

**OsRR12**	87116398

**OsRR13**	38637517

**OsRR13H**	87116400

**ZmRR1**	162463265

**ZmRR2**	162458152

**ZmRR3**	162458935

**ZmRR4**	195644464

**ZmRR5**	162459124

**ZmRR6**	162459272

**ZmRR7**	162459462

**ZmTCRR-1**	94966174

**ZmTCRR-2**	Not in the database

Protein names are taken from Ferreira and Kieber 2005, Ito and Kurata 2006, and Hirose et al. 2007; GI numbers are taken from the NCBI protein database. Note that OsRR13H corresponds to OsRR13 in Hirose et al. 2007.

### Expression of *ZmTCRR-1 *and *ZmTCRR-2 *in developing endosperms

**Figure 2:**
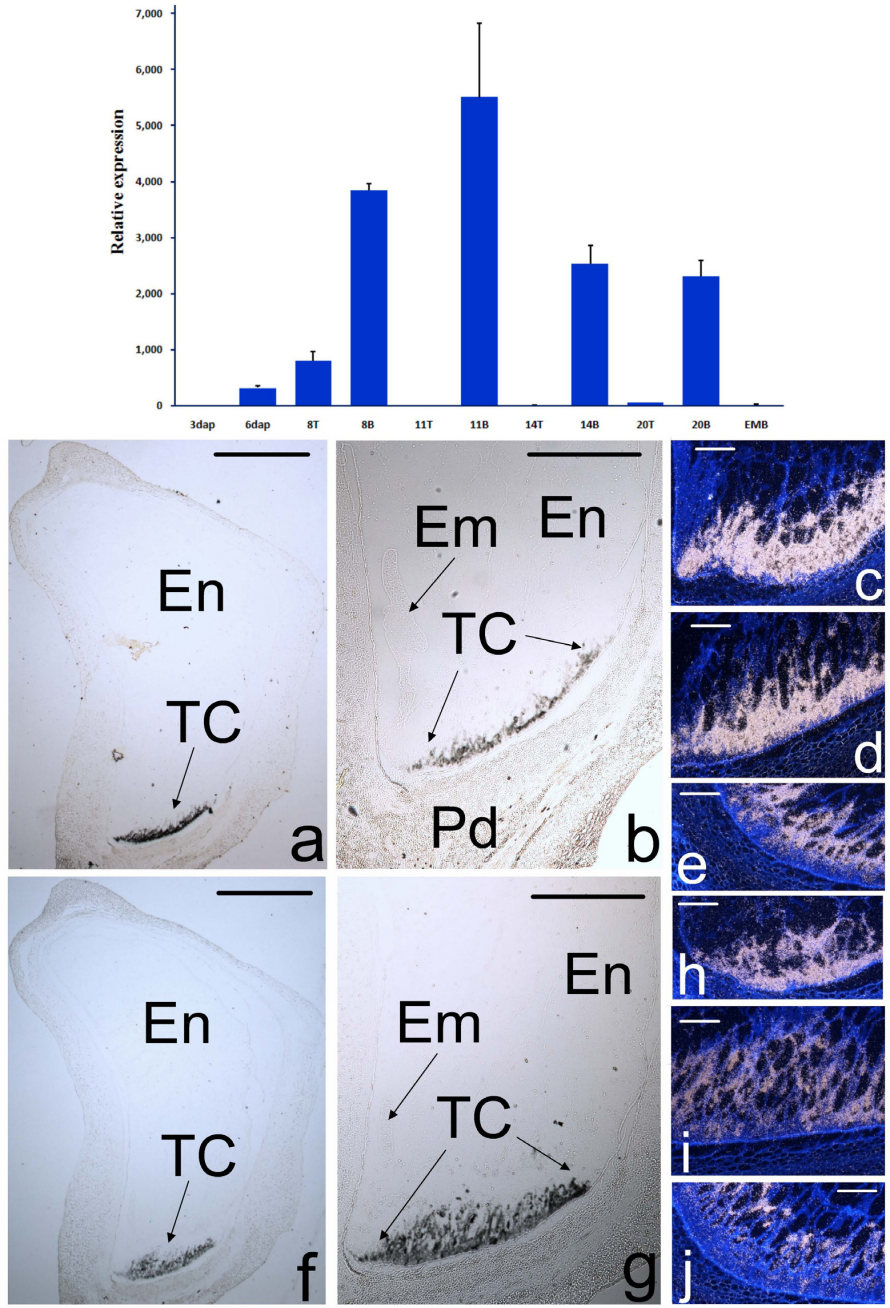
**Expression analysis of *ZmTCRR-2***. **Upper panel**, the graph represents the accumulation of the transcript along early development, relative to its level in the first time point tested (3 DAP). Numbers in the X-axis indicate days after pollination, EMB indicates embryo at 20 DAP, T and B indicate top (upper) and bottom (lower) half of the seed, respectively. **Lower panel**, Comparison of transcript accumulation sites of *ZmTCRR-1*(a to e) and *ZmTCRR-2 *(f to J) at 6 (c, h), 10 (a, b, d, f, g, i) and 14 (e, j) days after development, covering the transfer cells differentiation period. Images in a, b, f and g were taken in bright field using 1.25× (a, f) and 2.5 × (b, g) objectives. The condenser diaphragm was closed to increase contrast and visualize the unstained tissue. The hybridisation signal appears as a layer of black spots in the transfer cells. Images in c, d, e, h, i and j are dark field images, the signal is shown as white dots. Sections were counterstained with Calcofluor White to show the tissue structure. En, inner endosperm; Em, embryo; TC, transfer cells; Pd, pedicel. Scale bars represent 1 mm in a, b, f and g and 100 μm in c, d, e, h, i and j.

The expression of *ZmTCRR-1 *has been previously shown to be restricted to the transfer cells at the base of the kernel, in a reduced time frame during early development and peaking at 8-11 DAP (days after pollination) [25]. RT-PCR analysis of *ZmTCRR-2 *showed no expression in maize plant tissues other than the developing kernel (additional file 2). Within the seeds, an early peak was detected around 11 DAP and, contrary to the situation observed for *ZmTCRR-1*, a significant level of expression was observed until at least 20 DAP (Figure 2, upper panel). *In situ *hybridization experiments using the full length cDNA as a probe indicated that the expression of the gene was restricted to the transfer cell layer; no signal was detected in the embryo, inner endosperm or pedicel (Figures 2f, 2g). The signal remains confined to this area between 6 and 14 DAP (Figures 2h to 2j). The corresponding hybridizations with a *ZmTCRR-1 *probe (Figures 2a to 2e) are shown for comparison; these results show that the transfer cells express a pair of tissue-specific, developmentally controlled response regulators.

### Regulation of the expression of *ZmTCRR *genes

The promoter of *ZmTCRR-1 *has been previously shown to contain cis elements (called TC-boxes) responsive to a TC specific transcription factor, *ZmMRP-1 *[24]. A bioinformatic analysis of a 1700 base pair *ZmTCRR-2 *promoter fragment showed several motifs similar to the TC-box, TATCT at -266, -182 and -166 bp (inverted) from the translation start site. The 1700 bp upstream sequence was fused to the *UidA *reporter gene and cotransformed in onion epithelial cells with a constitutive *ZmMRP-1 *construct. After chromogenic detection, a positive transactivation result was obtained (additional file 3), indicating that *ZmMRP-1 *is a direct transactivator of *ZmTCRR-2*. Considering their identity as type-A response regulators, we tested the possible activation of both *ZmTCRR-1 *and *-2 *transcription by culturing developing kernels on media containing different hormones and performing either Northern Blot or qRT-PCR as detailed in Material and Methods. None of the treatments tested (kinetin, trans-zeatin riboside, naphthalene-acetic acid and gibberellic acid) produced a significant variation in the levels of transcript (not shown). We used a detached leaf system similar to the one described in references [2] and [28] to further check the effect of cytokinins on the expression of *ZmTCRR-1 *and *-2 *due to their structural similarity to TCS molecules. This system allowed the monitorization of the effect of the hormone treatment through the quantitation of well established inducible markers [28,29], which have not been described in the maize kernel to our knowledge. None of the ZmTCRRs was detected in the leaves, regardless of the treatment. In summary, our experiments did not provide any evidence for an involvement of cytokinin in the transcriptional activation of the ZmTCRRs (additional file 4).

### Expression pattern of candidate TCS upstream components

Two elements are positioned upstream of response regulators in the phosphorelay TCS pathways in plants, the sensory hybrid kinases and the intermediate histidine phosphotransfer proteins. In order to identify potential components of the TCS pathway in which *ZmTCRR-1*/-2 participate, we have analysed the expression pattern of these elements along seed development.

**Figure 3:**
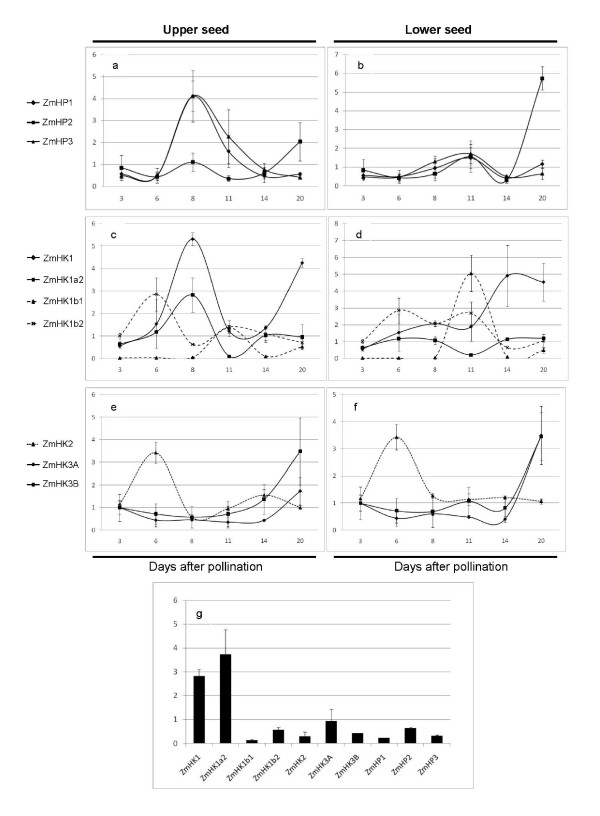
**Expression of TCS components along early seed development**. Results are the average and standard deviation of three replicates. Numbers in the X-axis indicate days after pollination. All values are relative to the expression of the housekeeping gene *ZmFKBP66*. Results are shown as lines and divided into upper (left panels) and lower (right panels) seed halves, except at 3 and 6 DAP, for which the sample represents the whole kernel. The expression in the embryo for each transcript at 20 DAP is represented as a single time point in a bar graphic (g).

Three HPs are currently known in maize [30], located on chromosomes 1, 2 and 4. A homology search did not show any additional loci in the maize genome. All three genes were found to be almost ubiquitously expressed (additional file 2). We found, however, differential expression patterns along seed development (Figures 3a and 3b, values normalised to the expression of the internal control *ZmFKBP66*; [31]). *ZmHP1 *and *3*, which are 88% identical in protein sequence, share also similar transcript kinetics, being already detectable at the earliest time point tested (3 DAP). Transcript levels peak at 8 DAP in the upper halves of the seeds and at 11 DAP in the lower ones. The overall variation in the tested interval is within one order of magnitude. *ZmHP2*, on the other hand, displayed a distinct expression pattern, showing stable low level accumulation of transcript until 20 DAP, when it raises, especially in the lower seed half, up to 18 times respective to the previous time point, and 5 to 9 times as compared to the other ZmHPs. This increase in expression did not extend to the embryo, which at 20 DAP expresses the 3 ZmHP genes at comparable levels (Figure 3g).

6 non ethylene receptors (NER) histidine kinases have been described in *Arabidopsis *[32] and rice [6]. We searched for additional loci to those described in [33] (*ZmHK1, 2 *and *3*, which displays two splicing alternatives) and [34] (*ZmHK1a2, 1b1 *and *1b2*) by homology searches against the maize sequence database http://www.maizesequence.org. The genome sequence analysis confirmed the presence of only these 6 separate HK loci in the current release of the maize genome sequence. qRT-PCR analyses of *ZmHK1, 1a2, 1b1, 1b2, 2, 3A *and *B *(loci located in chromosomes 5, 4, 1, 5, 3 and 5, respectively) showed ubiquitous expression for all members (additional file 2), in agreement with previous reports [33]. Along seed development, the level for the 7 histidine kinase transcripts was investigated using qRT-PCR (Figures 3c to 3f, values normalised to the expression of *ZmFKBP66*); *ZmHK1 *and *1a2*, which are 93% identical in amino acid sequence, peaked at 8 DAP in the upper part of the seed, with *ZmHK1 *being the major isoform at this point. Both transcripts decreased subsequently, but *ZmHK1 *rises again in the whole kernel at 20 DAP. *ZmHK1b1 *and *1b2*, in spite of being also very similar in their primary structure (93% identical in sequence, and approximately 60% to *ZmHK1 *and *1a2*), showed quite distinct expression kinetics. *ZmHK1b1 *was the least expressed of the ZmHK isoforms, except for a sharp, highly specific peak at 11 DAP, rising over 20 (top) and almost 100 times (bottom) from the previous time point, thus reaching expression levels comparable to the other transcripts at that stage. Remarkably, this expression pattern in the lower half of the seed almost replicates that previously reported for *ZmTCRR-1 *[25]. *ZmHK1b2*, on the other hand, was expressed at a higher basal level, showing two minor increases (less than 3-fold) at 6 and 11 days after pollination. *ZmHK2 *showed an early, 3-fold increase over the previous levels, peaking at 6 DAP in the whole seed and then decreased back to its original levels. Finally, the alternative splicing products of locus *ZmHK3*, maintained a stable expression level from 3 to 14 DAP, when they both started to increase. In the embryo at 20 DAP *ZmHK1 *and *1a2 *are the major hybrid kinase isoforms, *ZmHK1a2 *seems to accumulate preferentially in the embryo, given the low expression levels detected in the whole seed samples at 20 DAP (Figure 3g).

### Protein kinetics along development

The downstream interactors of type-A response regulators are currently unknown in most cases. There is however ample evidence to support their direct upstream interaction with histidine phosphotransfer proteins within the transduction route [30,35]. To study whether ZmTCRRs show coincidence with maize HPs in timing and location, polyclonal antibodies were raised against ZmTCRR-2, ZmHP1 and ZmHP2, the polyclonal anti-ZmTCRR-1 was already available [25]. Cross-reactivity tests using GST- and His-tagged proteins (additional file 5) proved that anti-ZmTCRR-1 recognizes both ZmTCRR peptides, whilst anti-ZmTCRR-2 is specific to this second protein, allowing its differential analysis. The anti-ZmHP1 serum recognizes ZmHP3 with lower affinity, which was expected due to their high sequence similarity, and ZmHP2, although in this case this cross-reactivity was only observed using relatively high amounts of recombinant proteins. This serum is thus referred from here on as anti-ZmHP1/3. The immunopurified anti-ZmHP2, however, does not react with the other phosphotransfer proteins, and allows the independent detection of this peptide.

**Figure 4:**
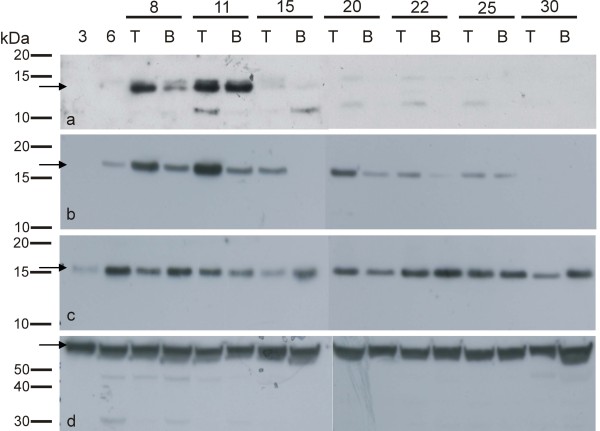
**Characterization of protein profiles along development**. Detection of native peptides in soluble extracts of 3 to 30 DAP kernels. T and B indicate upper (Top) and lower (Bottom) half of the kernel, respectively. **a **ZmTCRR-2; **b **ZmHP1 and 3; **c **ZmHP2; **d **ZmFKBP66, used as a loading control. The polypeptide with the predicted size is marked by an arrow in each case.

The ZmTCRR-2 peptide accumulation pattern reproduced almost identically that reported for ZmTCRR-1, being detectable since 6-8 DAP with a maximal accumulation at 11 DAP (Figure 4a). In spite of the lack of transcription in the upper seed half, the peptide was detectable in this area since the early developmental stages. Furthermore, the protein was only found in the upper seed halves at later developmental stages. The incomplete overlapping of protein and transcript accumulation patterns has been also reported in the case of *ZmTCRR-1 *[25] and very likely indicates that the proteins are exported towards the endosperm from the transfer cells where they are produced. The abrupt decay in protein concentration after 11 DAP very likely reflects the combination of reduced transcript levels at the transfer cells (Figure 2 upper panel) and dilution of the protein in the endosperm, where high amounts of storage proteins begin to accumulate after 11-14 DAP. Interestingly, the antisera detected two polypeptides within the expected size range (ca. 13 kDa). Considering the functional nature of this peptide, this double band may represent a phosphorylated form of the protein.

Concerning the detection of the phosphotransmitters, the anti-ZmHP1/3 antiserum detected a band of approximately 16 kDa, consistent with the expected sizes of ZmHP1 and 3 (16.4 and 16.2 kDa, respectively), which accumulated since 6 DAP and appeared to be more abundant in the upper seed half, consistently with the accumulation pattern observed for the transcripts. The band decreased in intensity along development and was no longer detectable by 30 DAP (Figure 4b). ZmHP2 was detected as a protein with an apparent size slightly smaller than the predicted 16.3 kDa, this may be explained by its high negative charge (-7.17) at the neutral pH of the electrophoresis system, which causes a faster than expected migration. The protein was present since 3 DAP all through the analysed interval, without significant differences between top and bottom halves within each time point (Figure 4c). No steep increase in peptide levels was observed, in contrast to the variation detected in the transcript accumulation at 20 DAP (Figure 3, panel b). This may be the result of the diluting effect of the reserve proteins being actively stored in the seed at that stage, although the existence of a post-translational regulation mechanism cannot be ruled out. Significantly, the absence of reacting proteins in several time point samples assayed with the anti-ZmHP1/3 antiserum indicates that this antibody cannot detect ZmHP2 at the concentrations found *in planta*. In summary, the three intermediate histidine phosphotransfer peptides are present in a time range which encompasses those of the ZmTCRRs.

### Protein cellular localization

**Figure 5:**
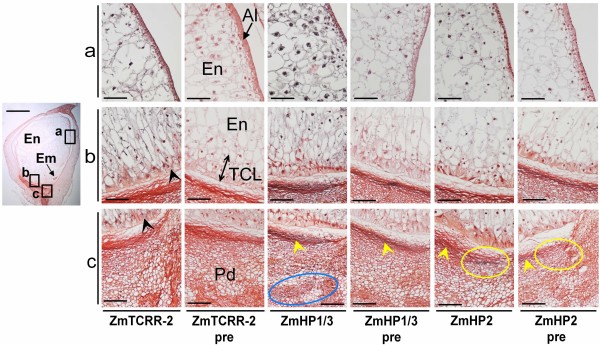
**Immunolocation of ZmTCRR-2 and ZmHPs on 10 DAP seeds**. Areas squared in the low magnification image are shown on the left after detection with anti-ZmTCRR-2, ZmHP1/3 and ZmHP2 antisera and preimmune sera as indicated below the images. The signal appears as dark/light grey precipitates depending on its intensity. **a **upper endosperm and aleurone show detection of ZmTCRR-2 and a strong signal from the ZmHP1/3 serum, while ZmHP2 is only weakly detectable. No unspecific signal is visible in the presera treated sections. **b **transfer cells and transmitting tissue. ZmTCRR-2 is present in the transfer cells (black arrowheads) and in the transmitting tissue located above them, as is the case for ZmHP1/3. Anti-ZmHP2, on the other hand, produces a weaker signal in this area, still clearly distinguishable from the preserum. **c **placento-chalaza and pedicel. A dark precipitate appears in the crushed placento-chalaza in all sections shown (yellow arrowheads), including presera, which indicates its unspecific nature. Apart from this, no signal from ZmTCRR-2 is detected in the area. Anti-ZmHP1/3, on the other hand, broadly decorates the pedicel specially at the vascular bundles (blue circle), while anti-ZmHP2 labels (apart from the unspecific signal mentioned above) two distinctive patches at the edges of the placento-chalaza. This area at the adgerminal end is shown in the panels for immune serum and preserum (yellow circles). En, inner endosperm; Em, embryo; TCL, transfer cell layer; Pd, pedicel. The scale bars represent 1 mm in the low magnification image and 100 μm in the other images.

The polyclonal antisera were used to detect the proteins at the cellular level by chromogenic immunolocation (Figure 5). The parallel use of preimmune sera allowed us to identify unspecific background from "sticky" areas, such as the placento-chalaza. At 10 DAP the anti-ZmTCRR-2 antibody produced a clear signal in the transfer cell area, extending into the endosperm tissue above it (ZmTCRR-2 panel b). The signal is visible in inner areas of the endosperm where no transcription is detected, as previously reported for *ZmTCRR-1 *[25], indicating a similar transport of the peptide into the endosperm storage tissue (ZmTCRR-2 panel a). No specific signal is found at the pedicel (ZmTCRR-2 panel c). Similar experiments performed with anti-ZmHP1/3 polyclonal serum labelled all endosperm compartments and the pedicel (ZmHP1/3 panels a, b, c). By contrast, ZmHP-2 was only weakly detectable at 10 DAP within the endosperm (ZmHP2 panels a and b); the protein accumulated, however, in two distinct areas at both ends of the pedicel vascular terminals (ZmHP2 panel c shows the one at the adgerminal side). At later stages (14 DAP) the clearest signal for this peptide is still concentrated in these areas (not shown). The corresponding preimmune panel shows no signal in this area.

### Protein-protein interaction experiments

**Figure 6:**
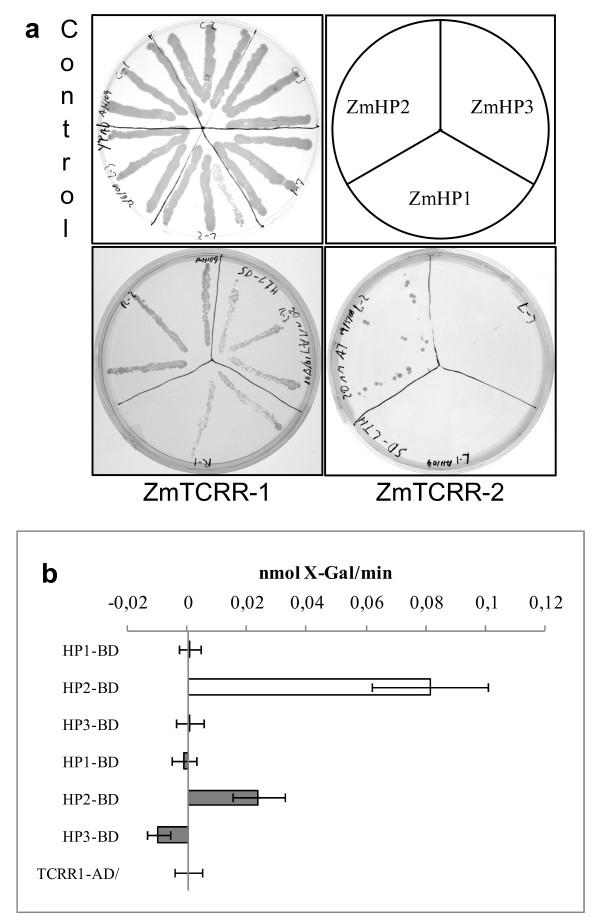
**Molecular interaction between ZmTCRRs and ZmHPs**. **a, **growth of AH109 yeast cells carrying ZmTCRRs-binding domain and ZmHPs-activation domain fusions on histidine-lacking medium. Upper left image, growth control on non selective medium after 10 days. Upper right image, distribution of ZmHPs-activation domain clones on the interaction plates. Lower left image, interaction plate for ZmTCRR-1 after 5 days; lower right image, interaction plate for ZmTCRR-2 after 10 days. **b, **quantification of interaction based on galactosidase activity in a 2-hybrid assay in YRG-2 cells. White bars, interaction of the ZmTCRR-1-activation domain fusion protein with ZmHP-binding domain fusions. Grey bars, interaction of the ZmTCRR-2-activation domain peptide with ZmHP-binding domain fusions. A yeast clone carrying only ZmTCRR-1-activation domain was used as to establish the background level. Each bar represents the mean and standard deviation of 3 independent experiments.

We tested the 3 ZmHPs for interaction with both ZmTCRR-1 and ZmTCRR-2 in a yeast-based 2-hybrid system. Using a GAL1-driven auxotrophy marker as a sensor for interaction, ZmTCRR-1 interacted with all three ZmHPs, supporting a faster growth in the ZmTCRR-1/ZmHP2 interaction. ZmTCRR-2, on the other hand, showed positive results in the interaction test only with ZmHP2, as neither ZmHP1 nor ZmHP3-carrying clones supported yeast growth even after extended culture (Figure 6a). A second, quantitative system based on fluorogenic galactosidase activity confirmed the ZmHP2 stable interaction, and indicated, on the basis of the quantification of galactosidase activity, that the interaction of ZmHP2 with ZmTCRR-1 is more stable than with ZmTCRR-2 (Figure 6b).

### Function of the *ZmTCRR-1 *promoter in *Arabidopsis*

**Figure 7:**
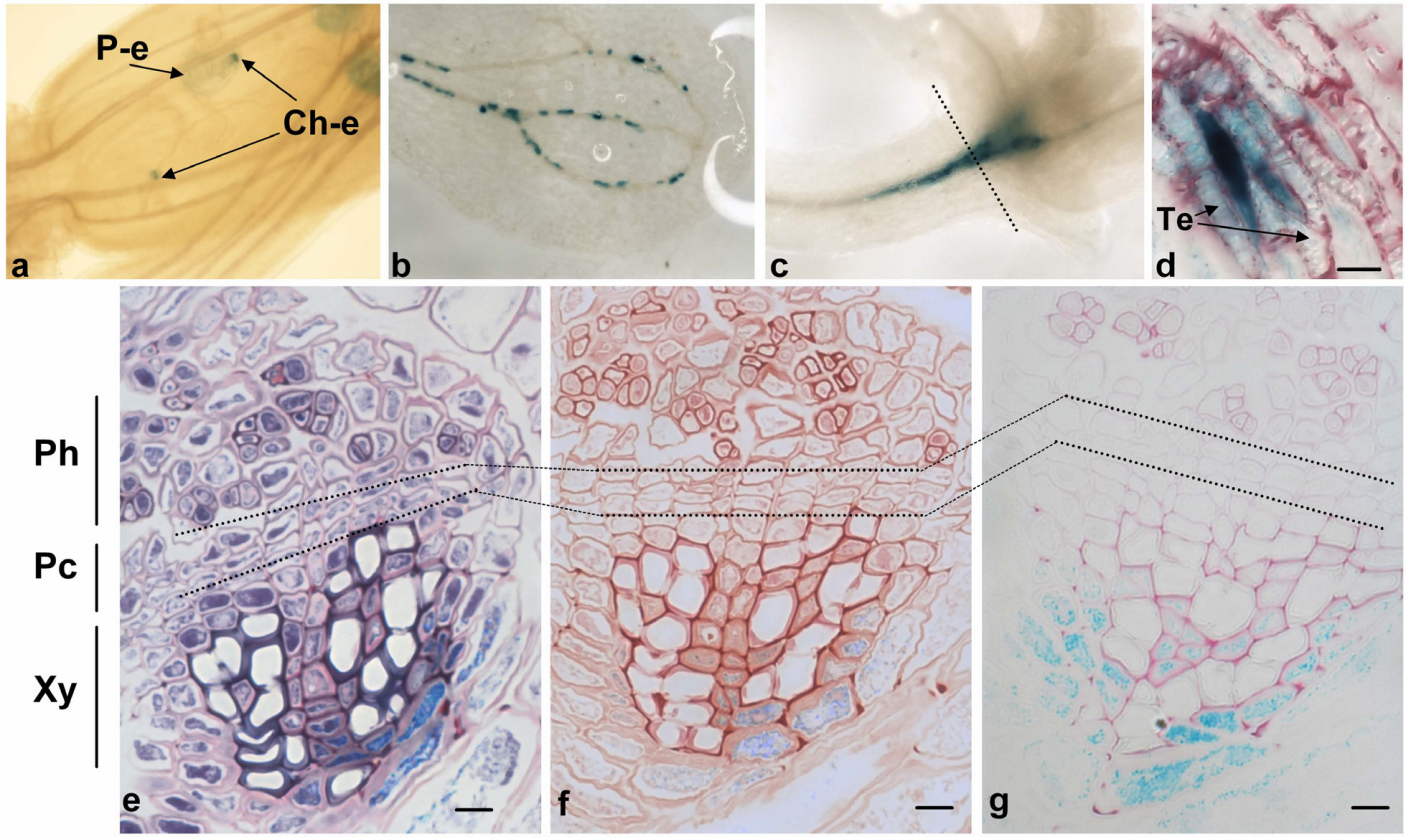
**Localization of GUS activity driven by the *ZmTCRR-1 *promoter in *Arabidopsis***. **a**, young developing seeds inside the siliques. The signal is concentrated on the chalazal endosperm (Ch-e) in contact with the funiculus, with a fade blue colour visible in the rest of the endosperm (peripheral endosperm, P-e). **b, **discontinuous staining of vessel bundles in the cotyledon. **c, **staining at the branching points of the vasculature below the apical meristem in 14 days old seedlings. **d, **longitudinal section showing one of the stained cells along the cotyledon vasculature and its association to the xylem tracheary elements (Te). **e, f, g, **transversal sections of vascular bundles at the intercotyledonary node (position indicated by a dashed line in d); sections were stained with toluidine blue (e), fuchsine (f) and ruthenium red (g). The primary cell wall appears pink in e and g, secondary cell wall appears dark blue in e and remains unstained in g. Cytoplasm is stained blue in e and red in f. GUS staining appears in all cases as a light blue signal. Ph, phloem; Pc, procambium; Xy, xylem. The scale bar in d, e, f and g is 20 μm.

We had previously obtained evidence indicating that the promoter of *ZmMRP-1*, the transcriptional regulator of *ZmTCRR-1 *and *-2*, was labelling nutrient exchange surfaces in *Arabidopsis *[36]. Here we analyzed in a similar way the promoter of *ZmTCRR-1*, the expression pattern of the *UidA *reporter gene was examined in 10 independent *A. thaliana *transgenic lines containing promoter:*UidA *constructs. The signal was transiently visible in the endosperm of developing seeds within young siliques, concentrating in the area identified as the chalazal endosperm (Figure 7a), a situation resembling that encountered in the maize basal endosperm. The GUS signal disappeared as seed maturation proceeded, being absent in imbibed and germinating seeds. In the seedling, positive GUS staining indicates promoter activation in isolated cells, associated to cotyledon and leaf venation (Figure 7b), and in vascular branching points immediately below the shoot apical meristem (Figure 7c). This signal was patent around 7 days after germination and became stronger around 14 days. Roots and other aerial parts lacked any signal throughout the plant's life cycle. Within the vascular bundles the signal localized to cells with a thickened primary wall, in close contact with xylem vessels. The staining was more intense in cells located in the internal side of the bundle, proximal to earliest developed vessel elements. (Figures 7d to 7g). The effect of different hormonal treatments on the promoter activation was tested. Neither naphthalene-acetic (NAA), indole-acetic acid (IAA), benzyl-aminopurine (BAP), gibberellic acid (GA3) nor ABA caused visible alteration on the location or intensity of the GUS signal when 7 day-old seedlings were cultured on them for 24 or 72 hours (not shown).

## Discussion

Since their first description [37], two component systems have been shown to take part in most of the transduction routes in bacteria and a number of sensing mechanisms in plants. Some functions have been attributed to particular molecules through mutant analysis [9,32,10], generally connected to cytokinin-mediated processes. In a previous paper we reported the isolation of a type-A response regulator specifically expressed in the basal endosperm transfer cell layer of the maize seed. This gene is transcribed under the control of a tissue-specific transcription factor in a restricted timeframe and the resulting peptide exported onto the starchy endosperm [25]. The protein (ZmTCRR-1) presents a number of unexpected features in a plant response regulator: it is short, with the regulatory domain encompassing the whole primary sequence and no C-terminal extension; its expression is not controlled by cytokinins, but developmentally regulated through a tissue specific transcription factor; it carries a D to H polymorphism affecting one of the amino acids in the active centre; and finally, and perhaps more strikingly, the protein accumulates in cells that do not transcribe the gene, suggesting a trans-cell operation of this signal transduction pathway.

In this work we have tried to identify other components of the TCS pathway in which *ZmTCRR-1 *participates. In addition, we have identified a second gene (*ZmTCRR-2*) with very similar characteristics to *ZmTCRR-1*. Both loci present a similar genomic structure and sequence identity of 62.4% at protein level. However, a possibly significant difference from a functional point of view is the conservation in ZmTCRR-2 of the canonical aspartic residue in position 13 (Figure 1b). Other than this, the similarity indicates a closer evolutionary ancestry between these sequences than to the rest of known maize response regulators [30]. In fact, a protein sequence comparison including type-A response regulators of *Arabidopsis thaliana*, *Oryza sativa *and *Zea mays *(Figure 1a) produces an independent branch where ZmTCRR-1 and ZmTCRR-2 cluster with four putative response regulators predicted in the rice genome [6,27]. These putative orthologues are also short (less than 140 residues) and contain a C-terminal motif not found in other type-A RRs in these 3 species, except ZmTCRR-1 and ZmTCRR-2. Furthermore, three of the four rice molecules (there are no data for OsRR13H) have been found to be expressed only in reproductive tissues.

The similarity between *ZmTCRR-1 *and *ZmTCRR-2 *extends to their expression pattern. *ZmTCRR-2 *transcript accumulates specifically at the BETL, peaking around 10 DAP. This similarity suggests a common regulatory mechanism. Furthermore, both transcripts can be detected in transfer tissue ectopically induced in the aleurone layer by the expression of *ZmMRP-1 *[38]. *ZmMRP-1 *is a transfer cell specific MYB-related, R1-type transcription factor, which has been shown to control a number of tissue specific genes (collectively known as BETL genes, reported in [39] and [20], including *ZmTCRR-1 *[25]. Significantly, the DNA binding domain of ZmMRP-1 contains the SHAQKYF motif, which is similar to the GARP motif found in type-B response regulators, responsible for the regulation of most type-A molecules [40]. The co-bombardment in onion of promoter-GUS fusions and the transcription factor under the control of a constitutive promoter shows that *ZmMRP-1 *is also able to induce transcription of *ZmTCRR-2 *(additional file 3). Collectively, these data point to *ZmMRP-1 *as a main transcriptional regulator of *ZmTCRR-2 *and *ZmTCRR-1*, and reinforce the role of this gene as a master regulator of transfer cell development. Although the existence of a yet unidentified type-B response regulator expressed in the transfer cells is possible, all the currently known members of this class seem to be ubiquitously expressed. Furthermore, the apparent lack of cytokinin-dependent transcriptional regulation in the *in vitro *cultures and detached leaf assays reinforces the hypothesis that these genes are not dependent on a standard type-B RR. Our data suggest an alternative model in which these type-A RRs would have been recruited by the *ZmMRP-1 *controlled pathway and subsequently adapted to participate in transfer cells' functions, which are not necessarily restricted to the processes occurring within the cells. This co-optation would be facilitated by the partial coincidence of the preferential recognition site of type-B RRs, AGATTC [40,5], and the core binding site of ZmMRP1 in BETL genes, in complementary orientation, TATCTC/GAGATA [24]. We have found similar sequences in the promoters of *ZmTCRR-1 *[25] and *ZmTCRR-2 *(this work), in the same relative positions than TC-boxes are located in BETL genes [22].

A further point of similarity between *ZmTCRR-1 *and *ZmTCRR-2 *is the polarized export of the corresponding proteins from the transfer cells into the endosperm, as shown by Western blot analyses and immunolocation with antisera differentiating both proteins. A number of proteins produced in the TC, or in the adjacent embryo surrounding region, are exported to the pedicel, in some cases to participate in defensive functions [21,41,42], which supports the active interfacial/exchange role of this tissue during seed development. An opposed polar signalling, from the transfer layer to the inner cell layers, is suggested by the differential appearance of these cells (known as transmitting tissue, [43]) as compared to the starchy endosperm cells. Transmitting cells are elongated along the pedicel-silk axis and facilitate the symplastic transport of metabolites towards the crown, where they are used to build storage polymers. Whether the primary role of ZmTCRRs is exerted in the transmitting tissue remains unknown, although examples of non cell autonomous functions based on protein movement can be found in the literature [44-46].

ZmTCRR-2 could be detected in the transfer cells, while ZmTCRR-1 is not [25]. This may reflect differential stabilisation mechanisms acting in the transfer cells, as those shown for phosphorilated vs. un-phosphorilated forms of ARR5 and 7 [47], or a simple difference in the antibodies' sensitivity in immunolocation. In any case the lack of the phosphorilation target aspartic in ZmTCRR-1 should be studied regarding this issue.

We have investigated the transcription of the other known components of TCS pathways (Figure 3), notably maize histidine kinases and histidine phosphotransfer proteins, along kernel development, trying to identify potential interactors of *ZmTCRR-1 *and -2. All these upstream components are almost ubiquitously expressed in the plant and have been shown to intervene in cytokinin signalling [33,30,48,49]. We found no significant quantitative variation (compared to the ZmTCRRs) for ZmHKs or ZmHPs expression during early seed development, except for the receptor *ZmHK1b1*, which showed an expression pattern remarkably similar to that of the ZmTCRRs. Further investigation of this receptor, including its tissue location, chemical ligand and downstream interactors is of clear interest, as it may clarify whether both ends of the transduction route are connected.

The analyses of the expression, protein accumulation and distribution within the kernel (Figure 3, Figure 4, and Figure 5) indicated that all three histidine phosphotransfer proteins co-localize with the ZmTCRRs within the kernel, and are thus possible interaction partners. The two hybrid results (Figure 6) pointed to ZmHP2 as the most probable interactor. However, local differences in protein concentration and availability of other interactors may determine the final partner in the kernel, or even variations along development. ZmHP2 accumulated preferentially in two distinctive patches under the collapsing placento-chalaza in opposite ends of the endosperm, and associated to vessel terminals (Figure 5), suggesting involvement with other TCS components, a common feature in histidine phosphotransfer proteins [50].

The relevance of cytokinins in the role of ZmTCRRs is merely speculative at this stage. However, the current data about the function of histidine phosphotransfer proteins point to cytokinins, and a number of papers show their effect on seed development in processes such as cell division and tolerance to heat stress [17] and maturation [51], and a careful regulation of their homeostasis along kernel development [52-54].

It must be noted that ZmTCRR-1 (lacking one of the canonical aspartic residues involved in the transfer of the phosphate group) interacts more strongly than ZmTCRR-2 with the phosphotransmitters (notably ZmHP2). The relevance of this feature *in planta *remains to be determined, but differences in the transfer rate or the turnover of the phosphorylation state have been proposed as a mechanism to finely tune the activity of response regulators in transmitting a changing signal [8,47].

We have previously shown [36] that the promoter of *ZmMRP-1 *responds to transport demanding situations (such as branching nodes, nematode feeding structures and base of developing fruits) in heterologous species. In this work we show that the *ZmTCRR-1 *promoter directs the expression of the *UidA *gene to the *Arabidopsis *developing seed and vascular tissues (Figure 7). The promoter activity within the seeds resembles that observed in maize for the *ZmTCRR *genes and the behaviour observed for the *ZmMRP-1 *promoter in *Arabidopsis*. The activity of the *ZmTCRR-1 *promoter in branching areas of the vascular system, particularly below the shoot apical meristem also resembles that reported for *ZmMRP-1 *[36] albeit the *ZmMRP-1 *promoter labelled more exchange surfaces.

A detailed study of GUS expressing cells shows staining of thick-walled cells associated to the xylem, interspersed and surrounding the earliest formed vessel elements within the bundle. The use of Ruthenium Red and Toluidine Blue stains, that allow detection of pectin and the differentiation of lignified and not lignified cell walls, respectively [55], show that these cells are surrounded by an engrossed middle lamella and primary wall, (Figures 7e, 7g), which identifies them as xylem parenchymatic cells. This cell type has been shown to participate actively in solute transport into and from the vessels, facilitating the retrieval of nutrients from xylem [56], response to osmotic variations [57] and participating in the modification of the cell wall in tracheary elements [58]. It's noteworthy that the *ZmTCRR-1 *activity is not continuous along the veins but limited to specific cells along the bundle, and is not visibly affected by growth on hormone-supplemented media (not shown).

## Conclusions

We have found two genes encoding type-A response regulators whose expression is cell type specific and developmentally regulated. We have shown that both molecules can be detected in the inner endosperm, and both are able to interact with known histidine phosphotransfer proteins. If ZmTCRRs are indeed able to respond to signals arriving from the mother plant they would provide developmental regulation to the response to an external stimulus, by which only the coincidence of the proper signal from the mother plant and the right differentiation/maturation stage from the interfacial BETL would transfer the message into the seed tissue. Whether these regulatory characteristics apply to the putative related rice molecules OsRR8, 12, 13 and 13H remains to be determined [6,27], but if so, it points to a conserved adaptation of this type of signalling pathways to reproductive specific functions.

## Methods

### Plant material

DNA and RNA samples were obtained from glasshouse-grown hybrid and A69Y maize plants. *Arabidopsis thaliana *var Col-0 was grown under long-day conditions (18 hours light/6 hours darkness, 70% humidity, 21°C/18°C).

### Cloning of the *ZmTCRR-1 *and *ZmTCRR-2 *promoters. Transformation into *Arabidopsis thaliana* or onion epithelial cells

The upstream sequence of *ZmTCRR-1 *(1200 bases upstream of the start codon) has been previously described and primers based on it were designed. 1700 bases of sequence upstream of the *ZmTCRR-2 *start codon were amplified with primers based on genomic information from the Maize Sequence Database http://www.maizesequence.org and KOD HiFi Taq polymerase (Novagen). All primers were designed with adequate AttB extensions, in order to clone the promoters into pDONR221 using Gateway BP technology (Invitrogen), producing pENTRY-TCRR1p and pENTRY-TCRR2p (primer sequences in Table 2, with Gateway adaptors underlined). A new recombination reaction allowed their transfer to pK2GWFS7,0 [59] producing a GFP:GUS reporter under the control of the *ZmTCRR-1 *or *ZmTCRR-2 *promoter. The *ZmTCRR-1 *construct was transformed into Col-0 plants using the method described in [60]. Ten independent transgenic events were produced. Two representative lines, bred to homozygosity, are shown to illustrate the expression of the construct. Activity of the promoter was localized by incubation of seedlings or plant tissues in buffer containing potassium ferro- and ferricyaniade (5 mM each), 50 mM sodium phosphate, 10 mM EDTA, 0.1% Triton X-100 and 1 mg/ml X-GLUC (Duchefa). To check the effect of hormone signalling on the promoter, homozygous transgenic seeds were plated on MS (Duchefa) plates for one week and then transferred to MS plates supplemented with either NAA, IAA, BAP, GA3 or ABA (5 μM) or unsupplemented. After 24 or 72 hours, 5 plants from each condition were stained for glucuronidase activity as above. For anatomical details, GUS-stained seedlings were embedded in LR White according to a protocol provided by Dr. Nicholas Harris (Dept. of Biological Sciences, University of Durham) and available at FTP directory/home/tair/Protocols/compleat_guide/athttp:// ftp://ftp.arabidopsis.org/home/tair/Protocols/compleat_guide/2_fix_and_embed.pdf, and sectioned at 0.5 μm thickness. The sections were counterstained with 0.01% Toluidine Blue in borax buffer, 2% fuchsine in water or Ruthenium Red 0.05% in water and photographed in an Axiophot microscope (Zeiss). The *ZmTCRR-2 *construct was used to transform onion epidermal cells (by particle bombardment) together with a ubiquitin promoter- *ZmMRP-1 *expression vector or an empty plasmid (pUBI-MRP and pUBI-NOS, described in [39]). The activity of the promoter was observed by incubation of the epidermal peelings in GUS buffer, as above.

**Table 2:** Sequences of the PCR primers used in this work.

CLONING OF CODING SEQUENCES	
TCRR2-GWS	AAAAAGCAGGCTCCATGGACACTATTGGTCCAC

TCRR2-GWAS	AGAAAGCTGGGTATCAAATATAGCTCAAGATACGAGG

HP1.3-GWS	AAAAAGCAGGCTCCATGTCTGCCGCGAACCAGC

HP1-GWAS	AGAAAGCTGGGTTACTTGTTGGGGGGAAAGC

HP3-GWAS	AGAAAGCTGGGTCACTTGCTGGGGGGAC

HP2-GWS	AAAAAGCAGGCTCCATGGCTGCCGCCGCGC

HP2-GWAS	AGAAAGCTGGGTTATTGTTGAGCCTGGATTTGC

AttB recombination motifs are shown underlined	

**CLONING OF *ZmTCRR-1 *AND *ZmTCRR-2 *PROMOTER**	

TCRR1p-GWS	AAAAAGCAGGCTAGCTTCATAGGATGATCCAC

TCRR1p-GWAS	AGAAAGCTGGGTGGACTAGCTAGACAAGCTC

TCRR2p-GWS	AAAAAGCAGGCTTAGTGTGCAATCGAAGCAACGG

TCRR2p-GWAS	AGAAAGCTGGGTAGATACTCTCCCACAACTTCC

AttB recombination motifs are shown underlined	

**qRT-PCR PRIMERS**	

HP1.3-QPCRS	AACACTTGCATTCAGTTCCGCG

HP1-QPCRAS	CCAGTACCTTGAGGCACCCATCTC

HP3-QPCRAS	CCAGTGTCTTGAGGCACCCATCTT

HP2-QPCRS	TCGTCACCCTCTTCTGCGACG

HP2-QPCRAS	CCACGATGGGCTGGTCAAGC

TCRR2-QPCRS	ATTCAAGTGACAATGGTGGAGGG

TCRR2-QPCRAS	CCAGGCATACAATAATCGGTCAGA

FKBP-QPCRS	GGGTGCTGTTGTTGAAGTCA

FKBP-QPCRAS	GCAATAACTTCCTCTTCATCG

HK1-QPCRS	GTGTGGCAGAGCATTGATTACAC

HK1-QPCRAS	TCACATACAAATACGGCAAGCTCA

HK1a2-QPCRS	GTGTGGCAGAGCATTGATAACGT

HK1a2-QPCRAS	ACTGCAAGCTCAATGCACTTCTCC

HK1b1-QPCRS	AATGGCAGTTCTCTAACCAGCACG

HK1b1-QPCRAS	TTTTGGGCAATCCAGGTGGACC

HK1b2-QPCRS	CGCTAATCAATGAAGTGCTTGACAG

HK1b2-QPCRAS	GATTCAAGATCCAACTTTCTGGC

HK2-QPCRS	CACAGGAGAAAGGACTGGAGTTGG

HK2-QPCRAS	GGATCGCCAATTAGTGTTTGTGG

HK3A-QPCRS	GTCATGCACCTGCAGTATTGG

HK3A-QPCRAS	TACATAAGTCACATTCATGCGGATT

HK3B-QPCRS	GCGATCGGCAGCATATTTGGAA

HK3B-QPCRAS	GCTGCGGAAACCAGACCAAAC

C4pepQPCR-S	GAGGCTCTGCAGAGAGAGATCC

C4pepQPCR-AS	CCATAGCGCATTTCGGCCTG

ZmRR2QPCR-S	GCGCAGCTCCAAGTACAGAGTGAC

ZmRR2QPCR-AS	TGTTCACGTCGGGGACCAGC

### Cloning of coding sequences into expression vectors, purification of recombinant proteins and polyclonal antibodies

The coding sequences of *ZmTCRR-2*, *ZmHP1*, *2 *and *3 *were amplified from seed cDNA using Gateway-adapted primers and recombined into pDONR221 (primer sequences in Table 2, with Gateway adaptors underlined). The resulting constructs (pENTRY-ZmTCRR2, pENTRY-ZmHP1,2 and 3 were further transferred into the bacterial expression vectors pDEST15 and pDEST17 (Invitrogen) for N-terminal GST-tagging or His-tagging, respectively. The constructs were transformed into *Escherichia coli *strain BL21A1, which expresses the recombinant proteins upon induction with L-arabinose. The recombinant peptides were isolated from the bacterial lysates using glutathion-sepharose 4B (GE Healthcare) and Ni-NTA agarose (Qiagen), following the manufacturer's instructions for native protein purification. The purity was checked by SDS-PAGE and approximately 5 mg of each GST-tagged protein (except GST-ZmHP3) were used to immunize rabbits along an 80-days period to obtain polyclonal sera (Immunostep SA, Salamanca, Spain). The serum against ZmHP2 was affinity purified using a His-tagged version of the protein as bait, and HiTrap Desalting and NHS-HP chromatography columns as indicated by the manufacturer (GE Healthcare). Additionally, an antiserum raised against 6 × His-ZmTCRR-1 (described in [25]) was used in this study.

### Two-hybrid analyses

The CDS of *ZmTCRR-1 *and *ZmTCRR-2 *were cloned in the prey vector pGBKT7, and *ZmHP1*, *2 *and *3 *were cloned in the bait vector pGADT7. All bait-prey combinations were transformed into competent AH109 yeast cells (MATa, trp1-901, leu2-3, 112, ura3-52, his3-200, gal4Δ, gal80Δ, LYS2::GAL1_UAS_-GAL1_TATA_-HIS3, MEL1 GAL2_UAS_-GAL2_TATA_-ADE2, URA3::MEL1_UAS_-MEL1_TATA_-lacZ). 3 separate colonies from each transformation were plated on SD-LWH plus 20 mM 3-amino-1,2,4-trizole (3-AT) and incubated at 30°C for 10 days. All media were prepared with 20 mg/L adenine sulphate, 2% glucose, 0.67% YNB (MP Biomedicals) and MatchMaker™ One Hybrid Library construction Drop Out amino acid mixes (Clontech). To further confirm the results, pENTRY vectors harbouring the coding sequences of *ZmTCRR-1*, *ZmTCRR-2*, *ZmHP1*, *2 *and *3 *were recombined into Gateway adapted versions of pAD-GAL4-2.1 Cam and pBD-GAL4 (Stratagene) to produce the bait TCRR1-AD and TCRR2-AD and prey HP1-BD, HP2-BD and HP3-BD constructs. All isolated constructs and bait-prey combinations were transformed into competent YRG-2 *Saccharomyces cerevisae *cells (Matα ura3-52 his3-200 ade2-101 lys2-801 trp1-901 leu2-3 112 gal4-542 gal80-538 LYS2::UAS_GAL1_-TATA_GAL1_-HIS3 URA3::UAS_GAL417 mers(*x*3)_-TATA_CYC1_-lacZ) and grown on the proper selection plates (SD-L for pAD constructs, SD-W for pBD constructs, SD-LW for cotransformations). 3 clones from each cotransformation plus 3 TCRR1-AD colonies (as a negative control) were grown in liquid, non selective media for 2 days at 30°C. Cells were pelleted and total proteins were extracted using YeastBuster™ Protein Extraction Reagent (Novagen). 15 μg total protein samples were used for enzymatic assays as described [61], with 4-Metilumbelliferyl-β-D-galactoside as the fluorogenic substrate. β-galactosidase activity was measured fluorimetrically in a Wallac Victor^2 ^1420 instrument.

### Expression analysis of TCS molecules

RNA samples from unpollinated flowers, upper and lower halves of 10 DAP seeds, leaves, roots, coleoptiles, silks and anthers were retro transcribed to cDNA using the SuperScript^® ^First-Strand Synthesis System (Invitrogen) to produce cDNA, following the manufacturer's instructions. 1 μL of each sample was used as template for PCR reactions using the qRT-PCR primers for *ZmTCRR-2 *and Biotools Taq DNA Polymerase. The expression of upstream TCS components in different maize tissues was studied by qRTPCR, using 7.5 ng of total RNA from each sample in duplicate reactions and the Express SYBR^® ^GreenER™ qPCR kit from Invitrogen, following the manufacturer's guidelines. Reactions were run on a RotorGene 6000 (Qiagen). For quantitative RT-PCR analysis of TCS components in the seed, the SuperScript^® ^III Platinum^® ^One-step qRT-PCR kit was used (Invitrogen). Each reaction (a triplicate was run per sample) contained 15 ng of total RNA and was run on an ABI 7000 instrument, using the cycling and detection conditions suggested by the kit manufacturer. To test the effect of hormonal treatments on *ZmTCRR-1 *and *-2 *expression, ears were collected at 6 DAP and the sheath was removed under sterile conditions. Using forceps and a scalpel, pieces of ear with 4-6 kernels attached were dissected and placed on culture medium prepared as described in [62], supplemented with either mock, kinetin (10 μM), trans-zeatin riboside (10 μM), GA_3 _(30 μM) or NAA (5 μM). Seeds were collected at 6, 24 and 42 hours of culture and RNA was extracted and analysed either by Northern Blot (with a full length *ZmTCRR-1 *cDNA probe) or qRT-PCR (with *ZmTCRR-2 *specific primers, see Table 2). In order to further evaluate the effect of cytokinins, the third and fourth leaves of three week-old plants were excised and submerged by their base in distilled water for 12 hours to simulate nitrate deprivation. Mock-treated leaves were then kept in water for 6 additional hours, while TZR-treated plants were incubated for the same time in distilled water containing 5 μM trans-zeatin riboside. RNA was extracted and the levels of *ZmTCRR-1*, *ZmTCRR-2*, *C4Ppc1 *(C4 phosphoenol pyruvate carboxylase 1) and *ZmRR2 *were measured by qRTPCR as described above. Each measurement was done in quadruplicate (2 biological and 2 technical replicates). The PCR primers (see Table 2) were designed based on maize cDNA and genomic sequences from the Maize Sequence Database. At least one primer in each set was designed spanning an intron splicing junction. All data, after evaluation of specificity by analysis of the dissociation curves, were analysed using the Genex Gene Expression Analysis Excel macro (BioRad) and are shown as values relative to the expression of the ubiquitously expressed gene *ZmFKBP-66 *[21].

### Western Blot experiments

Kernels at 3 and 6 DAP, and the top and bottom halves of 8-, 11-, 15-, 20-, 22-, 25- and 30-DAP seeds were ground in liquid nitrogen. The powder was extracted at 4°C with 0.1 M phosphate buffer containing a cocktail of protease inhibitors (Roche). The supernatant was mixed with 1/4 volume of 4 × LDS-containing protein loading buffer (Invitrogen) and 1/10 volume of 0.4 M DTT, heated at 70°C for 10 minutes. Several replicates were then separated on 12% pre-cast NuPAGE Novex gels (Invitrogen, Carlsbad, CA, USA) using a 2-(N-morpholine)-ethanesulphonic acid (MES) buffer system. Transference to polyvinylidene fluoride filters (Millipore, Bedford, MA, USA) was performed using the XCell II Blot Module apparatus and buffers (Invitrogen). The filters were then subjected to immunodetection with the antisera against the different proteins of interest (anti-ZmTCRR-2 diluted 1/1000, anti-ZmHP1/3 diluted 1/2000, anti-ZmHP2 diluted 1/400 and anti-FKBP66 diluted 1/5000). A goat anti-rabbit monoclonal antibody conjugated to peroxidase was used at 1/40000 dilution and the immunocomplexes were detected by incubation with a chemiluminiscent substrate (Super Signal West Pico Chemiluminiscent Substrate, Pierce) and autoradiography. For cross-detection controls, purified recombinant proteins (5 ng of 6×His-TCRR-1, GST-TCRR-1 and GST-TCRR-2) were electrophoresed and transferred in duplicate as described above. The anti-ZmTCRR-1 and -2 sera were used at 1/500 and 1/1000 dilutions, respectively, and detected as described above. Serial dilutions (2 to 0,02 ng) of recombinant GST-HP1,-2, -3 and -MRP1 (as a control for GST antigenicity) were spotted in duplicate on nitrocellulose and detected as above with anti-ZmHP1/3 and anti-ZmHP2 at 1/500 dilutions.

### Hybridization and Immunolocation

A69Y seeds at 5-20 DAP were fixed in paraformaldehyde/glutaraldehyde, dehydrated in an ethanol series, embedded in ParaPlast plus embedding medium (Sigma), and cut into 8 μm sections as described in [22]. For *in situ *hybridization, slides were hybridized essentially as described in [63]. 35S-labeled RNA was synthesized from pENTRY-ZmTCRR1 and pENTRY-ZmTCRR2 templates using an in vitro transcription kit (Roche Diagnostics). Slides were exposed for 2 to 4 days at 4°C using LM-1 silver grain emulsion (Amersham). After developing, sections were stained with Calcofluor White (0.01%) for 15 min and washed with water for 3 × 10 min each. Immunolocalization was performed using each preserum-serum pair at equivalent dilutions on 10 DAP sections. The slides were rehydrated in an ethanol series and endogenous peroxidase activity was deactivated by incubation in 0.3% hydrogen peroxide. Sections were then blocked with 2% normal donkey serum and incubated with the antisera or presera (ZmTCRR-2, diluted 1/150; ZmHP1/3, diluted 1/400; ZmHP2, diluted 1/80). The slides were washed twice in PBT and incubated in biotin-conjugated anti-rabbit goat antibody at 1/750 (Sigma). After two more washes, an Extravidin-peroxidase (Sigma) solution at 1/800 was added to each slide and further incubated. Finally, the slides were washed twice and incubated in SIGMAFAST™ DAB with Metal Enhancer (Sigma) until the precipitate was clearly visible on the sera slides. Reactions were then stopped by intensive washing in water. Sections were counterstained in 0.1% safranin. All microphotographies were taken using an Axiophot microscope (Zeiss).

## Authors' contributions

LMM performed the gene expression and protein analysis, prepared expression and reporter constructs, purified the recombinant proteins, performed the plant transformation and anatomical studies and drafted the manuscript. JR conducted the two-hybrid analysis. EG carried out the immunolocation experiments in maize kernels. GB and WP screened the cDNA library for *ZmTCRR-1 *related genes. GH performed the in situ hybridization experiments and collaborated in the immunopurification of anti-ZmHP2. All authors revised the draft, read and approved the final manuscript.

## Supplementary Material

Additional file 1 **Synteny of ZmTCRRs genomic regions to rice and sorghum**. Using the synteny tool available in the Maize Database we have determined the existence of syntenic regions in the genomes of *Oryza sativa *and *Sorghum bicolor*. Upper panels, co-linear arrangement of maize chromosome 4 (harbouring *ZmTCRR-1*) and the corresponding rice (left) and sorghum (right) chromosomes. Lower panels, co-linear arrangement of maize chromosome 1 (harbouring *ZmTCRR-2*) and the corresponding rice (left) and sorghum (right) chromosomes. Red circles and bars indicate the location of syntenic regions around the *ZmTCRR *loci. Click here for file

Additional file 2 **Expression of TCS components in different maize tissues**. Gel image, 1 μL of cDNA from unpollinated flowers (U), top (T) and bottom (B) halves of 10 DAP seeds, leaves (L), roots (R), coleoptyles (C), anthers (A) and silks (S) was used for PCR detection of expression of *ZmTCRR-2*. The reactions (plus the corresponding negative control, -) were run on 1.5% agarose gels. *ZmFKBP66 *amplification was used as normalization control. Lower panels, expression of TCS components in several maize tissues. Results are the average and standard deviation of two replicates. All values are relative to the expression of the corresponding gene in leaves and normalised to the housekeeping gene *ZmFKBP66*. Samples are unpollinated flowers (U), lower half of 11 DAP seed (B), aleurone at 25 DAP (Al), leaves (L), roots (R), coleoptyles (C), anthers (A) and silks (S). Click here for file

Additional file 3 **Transactivation of the ZmTCRR-2 promoter by *ZmMRP-1***. Onion epithelial cells were cotransformed with a *ZmTCRR-2prom:UidA *construct and either a constitutive Ubiquitin promoter:*ZmMRP-1 *(*pUbi-MRP*) or a mock Ubiquitin promoter:empty vector (p *Ubi-NOS*), and promoter activation was detected by GUS staining. *pUbi-MRP/ZmTCRR-1prom:UidA *and *35S-CMVprom:UidA *transformations were performed as controls. Each experiment was done in triplicate. **a, **representative images of bombardment results, showing the most densely stained areas. **b, **total transformed cells in each experiment (including all replicates). Click here for file

Additional file 4 **Effect of cytokinins on ZmTCRRs expression**. Detached leaves of wild type, three weeks-old plants were treated with trans-zeatin riboside (5 μM) and the transcript levels of both ZmTCRRs and 2 cytokinin inducible markers were determined. No signal was detectable for any of the TC transcripts, while *C4Ppc1 *and *ZmRR-2 *responded with 3-fold and over 600-fold inductions upon treatment, respectively. Transcript levels are normalized to the expression of *ZmFKBP66 *and referred to the mock sample. Mock, distilled water; TZR, 5 μM trans-zeatin riboside; ND, not detected. Click here for file

Additional file 5 **Cross-reaction tests for the sera used in this work**. **a, **His- or GST-tagged ZmTCRR-1 and -2 were separated by SDS-PAGE, transferred to PVDF and detected with anti-ZmTCRR-1 (right panel) or anti-ZmTCRR-2 (left panel). Lanes 1 and 4, 6×His-TCRR-1 (16,67 kDa); lanes 2 and 5, GST-TCRR-2 (41,53 kDa); lanes 3 and 6, GST-TCRR-1 (41,55 kDa). The anti-ZmTCRR-2 serum (generated against GST-TCRR-2) strongly detects GST-TCRR-2 and weakly GST-TCRR-1, probably due to recognition of the GST tag, as 6×His-TCRR-1 is not detected. Anti-ZmTCRR-1, on the other hand, reacts to all three recombinant proteins. A scheme of the PageRuler Unstained Protein ladder (Fermentas) is shown at the left for size determination. **b, **serial dilutions of GST-tagged ZmHP1,-2, -3 and -MRP1 (2 to 0,02 ng) were spotted on nitrocellulose and reacted with anti-ZmHP1/3 (left) or anti-ZmHP2 (right) sera. While the anti-ZmHP1/3 serum detects all three histidine phosphotransfer proteins and the GST tag attached to ZmMRP-1, anti-ZmHP2 is specific for ZmHP2. Click here for file
