# Supplementary material for: Atypical response regulators expressed in the maize endosperm transfer cells link canonical two component systems and seed biology
Source: BMC Plant Biol. 2010 May 7;10:84. doi: 10.1186/1471-2229-10-84 (PMC3017813; doi:10.1186/1471-2229-10-84)

ZmTCRR-1 genomic region sinteny to rice and sorghum

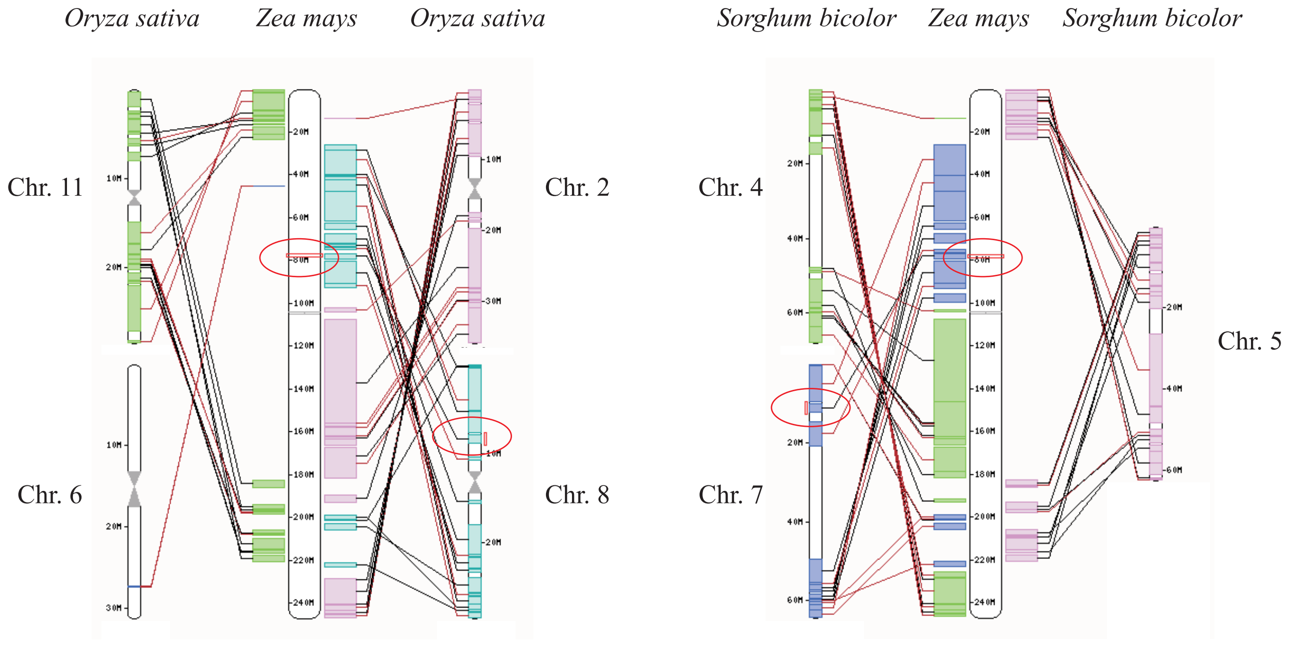

ZmTCRR-2 genomic region sinteny to rice and sorghum

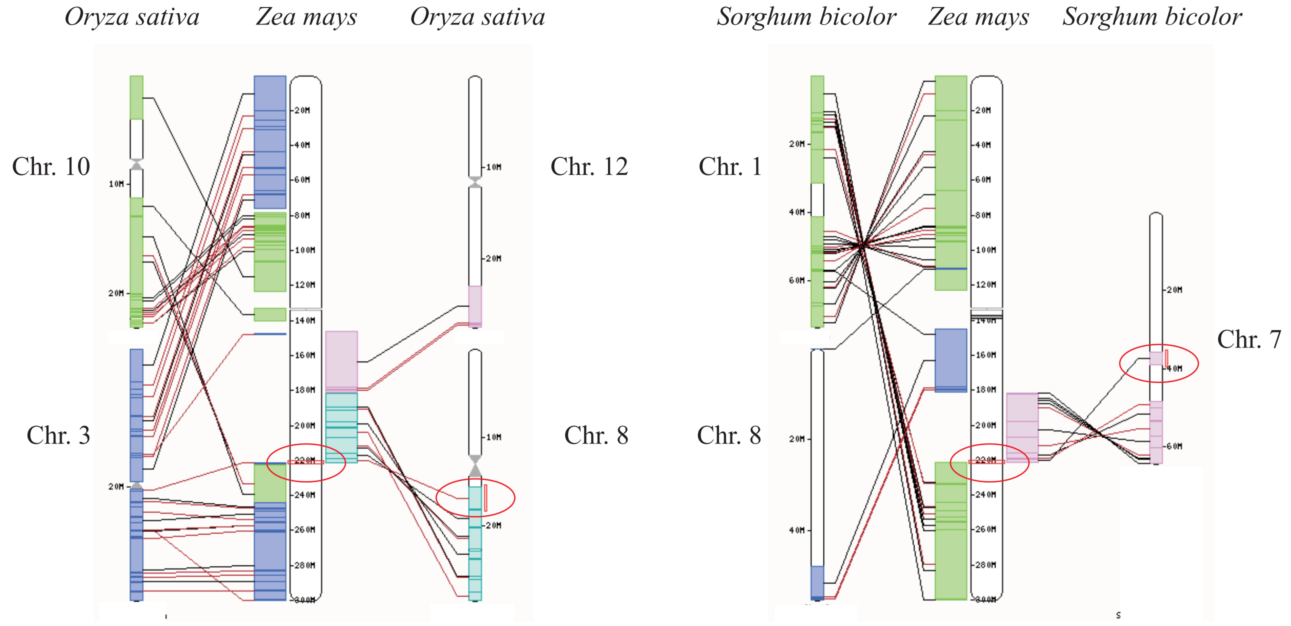

Supplement: Additional file 1 — Synteny of ZmTCRRs genomic regions to rice and sorghum. Using the synteny tool available in the Maize Database we have determined the existence of syntenic regions in the genomes of Oryza sativa and Sorghum bicolor. Upper panels, co-linear arrangement of maize chromosome 4 (harbouring ZmTCRR-1) and the corresponding rice (left) and sorghum (right) chromosomes. Lower panels, co-linear arrangement of maize chromosome 1 (harbouring ZmTCRR-2) and the corresponding rice (left) and sorghum (right) chromosomes. Red circles and bars indicate the location of syntenic regions around the ZmTCRR loci. [file 1471-2229-10-84-S1.PDF]
