# Supplementary figures and images for: Atypical response regulators expressed in the maize endosperm transfer cells link canonical two component systems and seed biology
Source: BMC Plant Biol. 2010 May 7;10:84. doi: 10.1186/1471-2229-10-84 (PMC3017813; doi:10.1186/1471-2229-10-84)

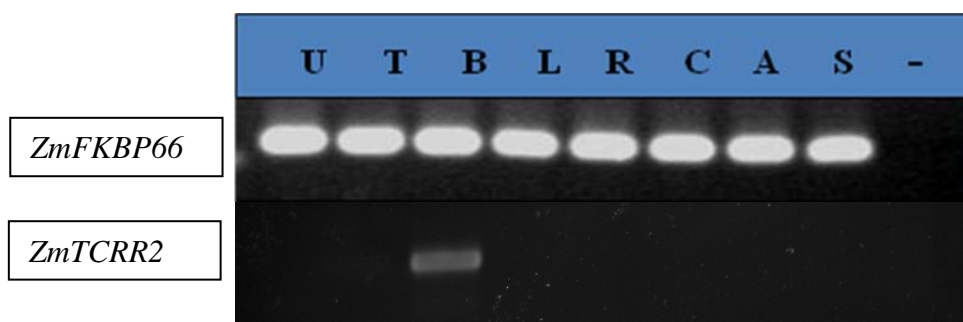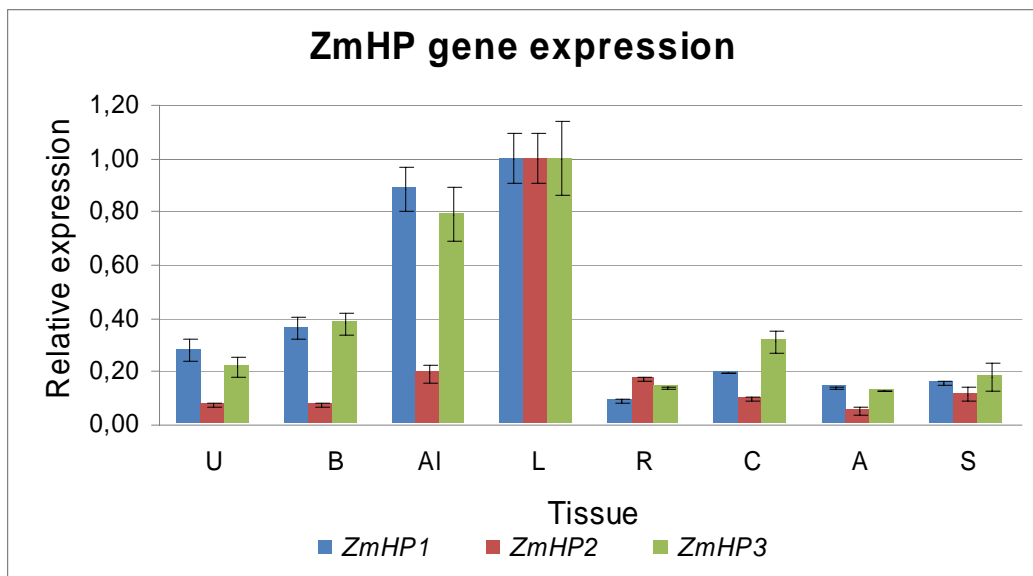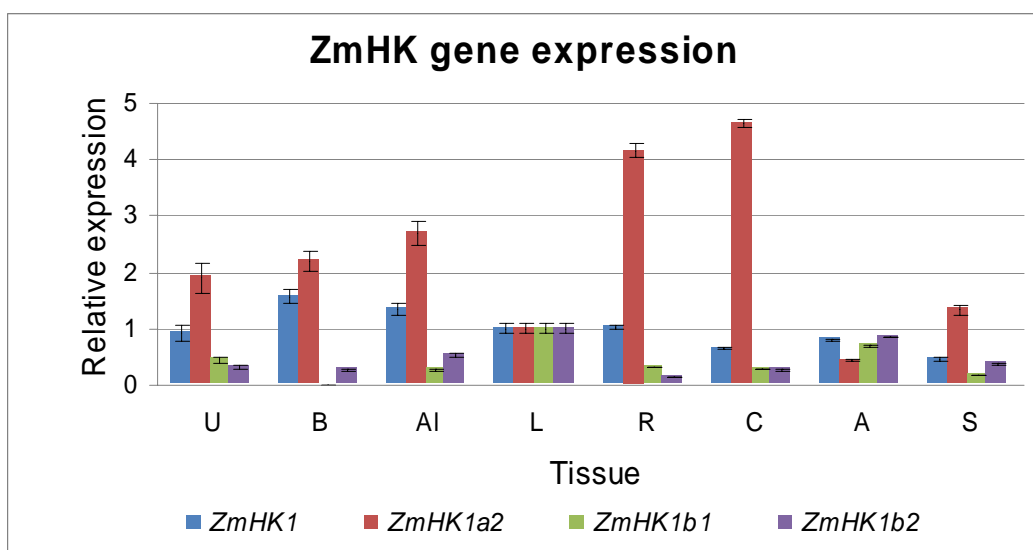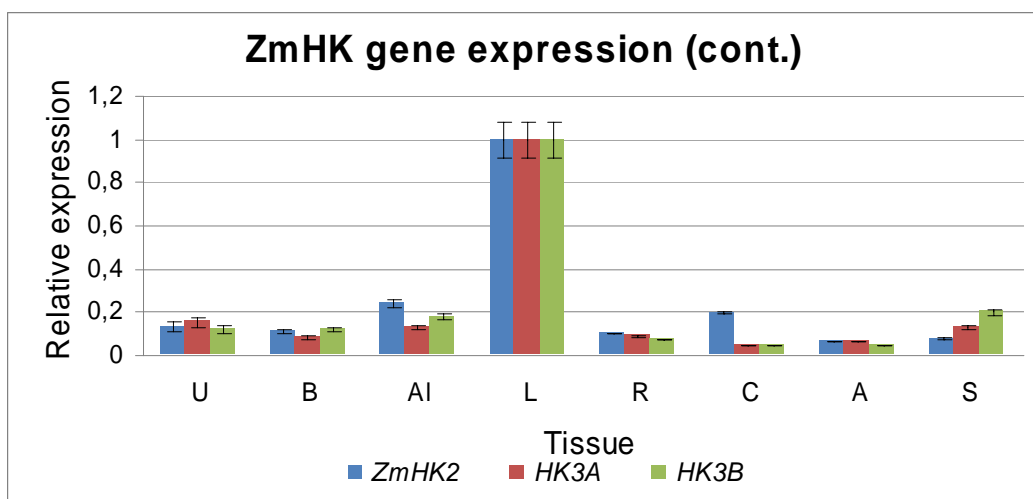

Supplement: Additional file 2 — Expression of TCS components in different maize tissues. Gel image, 1 μL of cDNA from unpollinated flowers (U), top (T) and bottom (B) halves of 10 DAP seeds, leaves (L), roots (R), coleoptyles (C), anthers (A) and silks (S) was used for PCR detection of expression of ZmTCRR-2. The reactions (plus the corresponding negative control, -) were run on 1.5% agarose gels. ZmFKBP66 amplification was used as normalization control. Lower panels, expression of TCS components in several maize tissues. Results are the average and standard deviation of two replicates. All values are relative to the expression of the corresponding gene in leaves and normalised to the housekeeping gene ZmFKBP66. Samples are unpollinated flowers (U), lower half of 11 DAP seed (B), aleurone at 25 DAP (Al), leaves (L), roots (R), coleoptyles (C), anthers (A) and silks (S). [file 1471-2229-10-84-S2.PDF]

**a***pUbi-NOS/ZmTCRR-2:Uida**pUbi-MRP/ZmTCRR-2:Uida*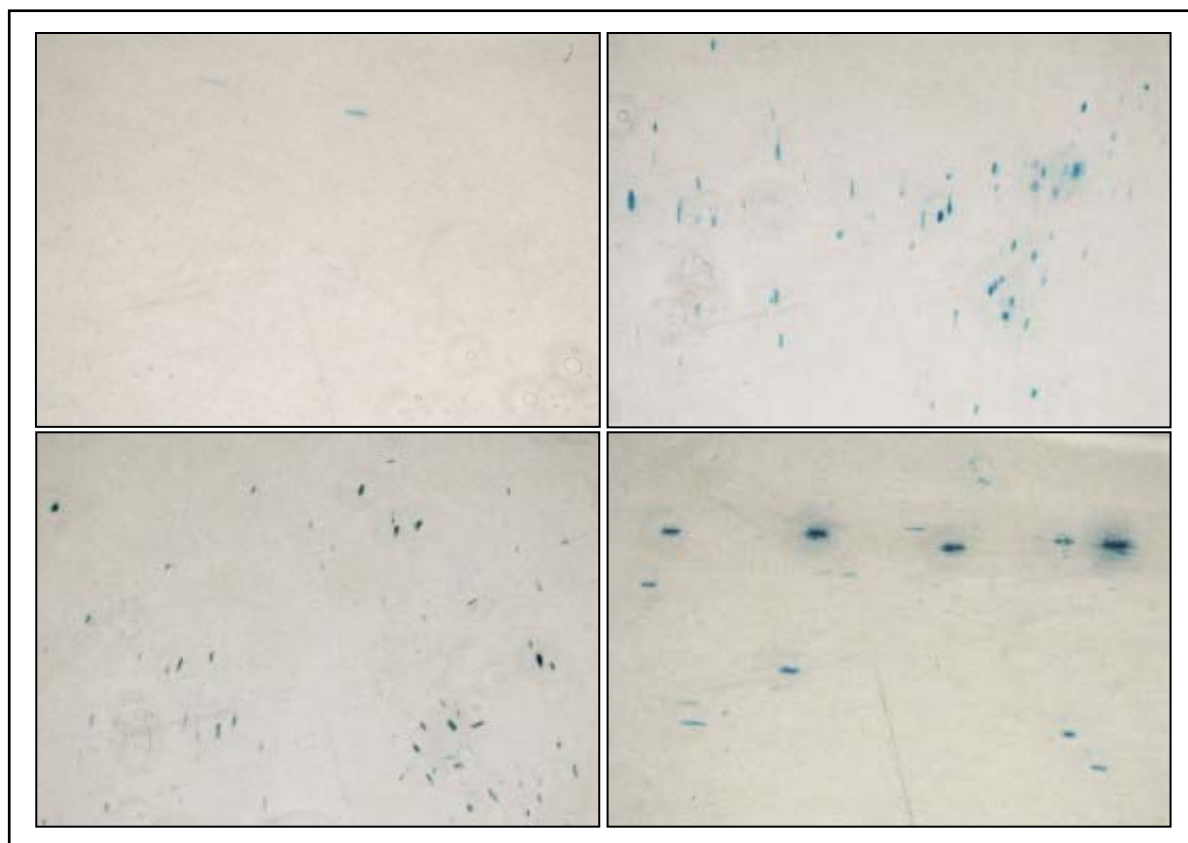*pUbi-MRP/ZmTCRR-1:Uida**35SCMV:Uida***b**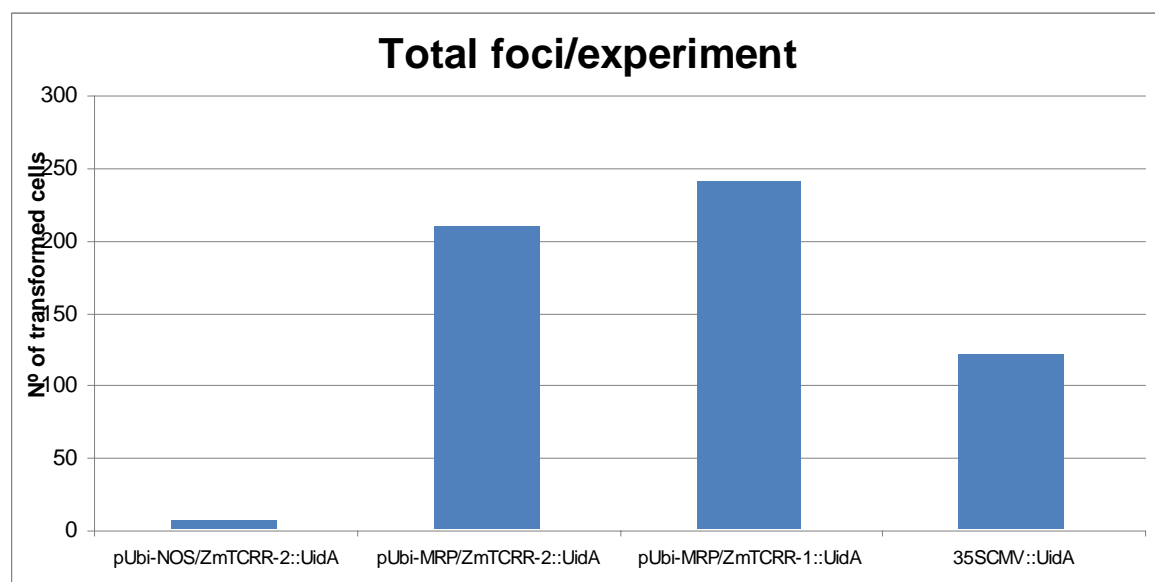

Supplement: Additional file 3 — Transactivation of the ZmTCRR-2 promoter by ZmMRP-1. Onion epithelial cells were cotransformed with a ZmTCRR-2prom:UidA construct and either a constitutive Ubiquitin promoter:ZmMRP-1 (pUbi-MRP) or a mock Ubiquitin promoter:empty vector (p Ubi-NOS), and promoter activation was detected by GUS staining. pUbi-MRP/ZmTCRR-1prom:UidA and 35S-CMVprom:UidA transformations were performed as controls. Each experiment was done in triplicate. a, representative images of bombardment results, showing the most densely stained areas. b, total transformed cells in each experiment (including all replicates). [file 1471-2229-10-84-S3.PDF]

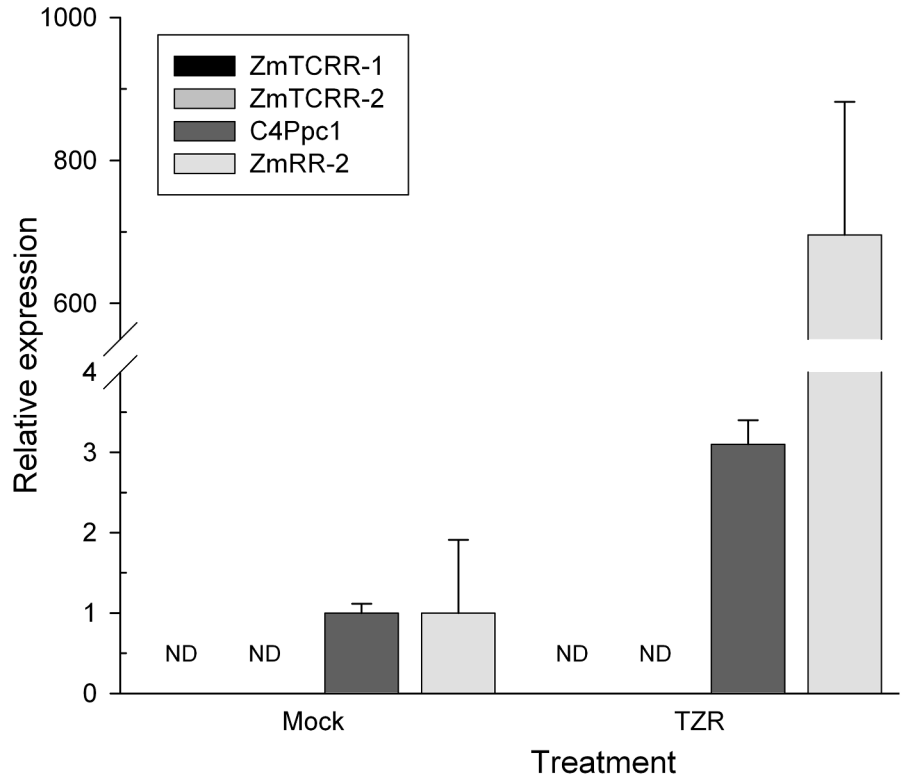

Supplement: Additional file 4 — Effect of cytokinins on ZmTCRRs expression. Detached leaves of wild type, three weeks-old plants were treated with trans-zeatin riboside (5 μM) and the transcript levels of both ZmTCRRs and 2 cytokinin inducible markers were determined. No signal was detectable for any of the TC transcripts, while C4Ppc1 and ZmRR-2 responded with 3-fold and over 600-fold inductions upon treatment, respectively. Transcript levels are normalized to the expression of ZmFKBP66 and referred to the mock sample. Mock, distilled water; TZR, 5 μM trans-zeatin riboside; ND, not detected. [file 1471-2229-10-84-S4.PDF]

**a**

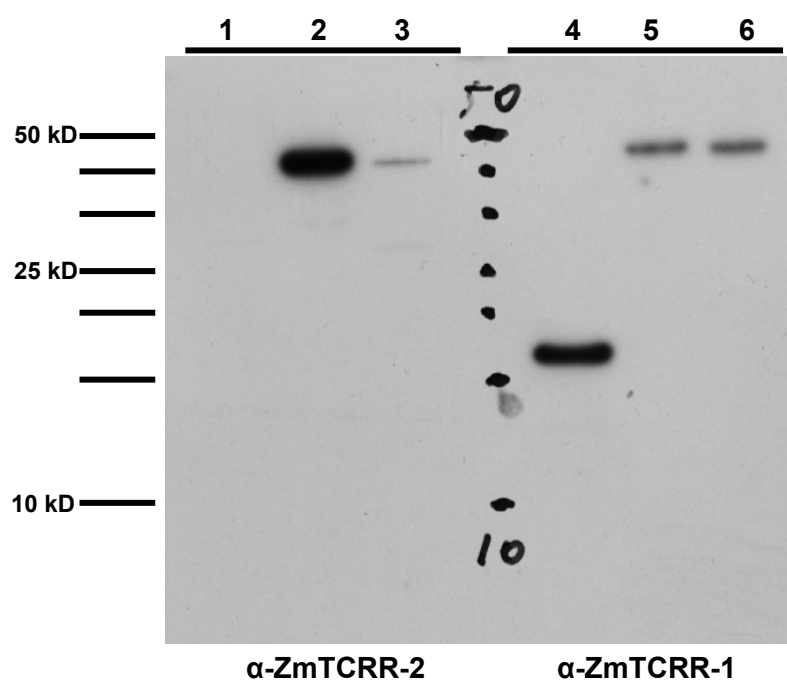

**b**

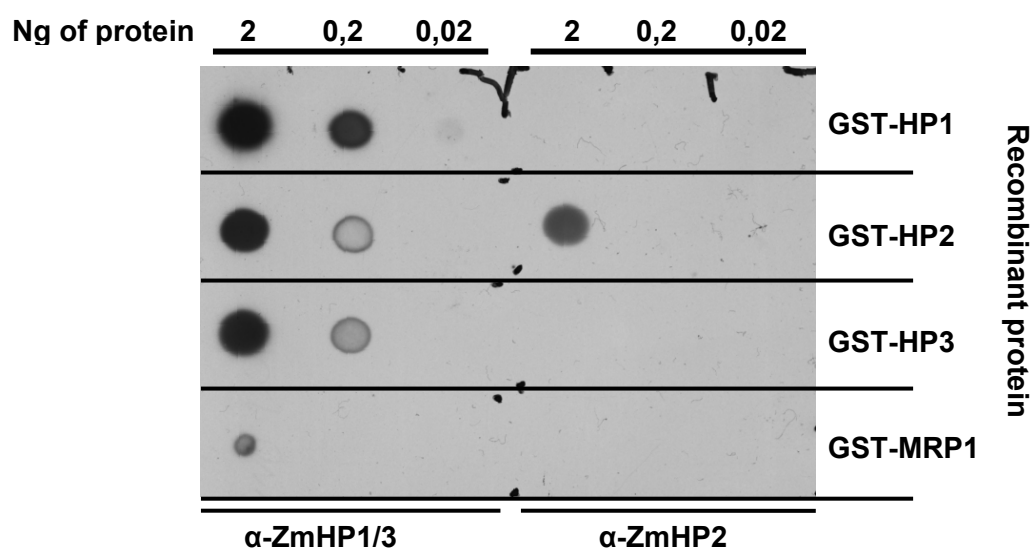

Supplement: Additional file 5 — Cross-reaction tests for the sera used in this work. a, His- or GST-tagged ZmTCRR-1 and -2 were separated by SDS-PAGE, transferred to PVDF and detected with anti-ZmTCRR-1 (right panel) or anti-ZmTCRR-2 (left panel). Lanes 1 and 4, 6×His-TCRR-1 (16,67 kDa); lanes 2 and 5, GST-TCRR-2 (41,53 kDa); lanes 3 and 6, GST-TCRR-1 (41,55 kDa). The anti-ZmTCRR-2 serum (generated against GST-TCRR-2) strongly detects GST-TCRR-2 and weakly GST-TCRR-1, probably due to recognition of the GST tag, as 6×His-TCRR-1 is not detected. Anti-ZmTCRR-1, on the other hand, reacts to all three recombinant proteins. A scheme of the PageRuler Unstained Protein ladder (Fermentas) is shown at the left for size determination. b, serial dilutions of GST-tagged ZmHP1,-2, -3 and -MRP1 (2 to 0,02 ng) were spotted on nitrocellulose and reacted with anti-ZmHP1/3 (left) or anti-ZmHP2 (right) sera. While the anti-ZmHP1/3 serum detects all three histidine phosphotransfer proteins and the GST tag attached to ZmMRP-1, anti-ZmHP2 is specific for ZmHP2. [file 1471-2229-10-84-S5.PDF]
